# Predicting Occupational Outcomes for Individuals with ADHD: The Role of Hyperactivity/Impulsivity and Executive Functioning

**DOI:** 10.1007/s10926-024-10259-y

**Published:** 2024-12-12

**Authors:** Elizabeth S. M. Chan, Joshua M. Langberg

**Affiliations:** Graduate School of Applied and Professional Psychology, Rutgers University, Piscataway, NJ USA

**Keywords:** Attention deficit/hyperactivity disorder, Occupational functioning, Work, Time management, Organization, Motivation

## Abstract

**Purpose:**

The present study adds to the literature by evaluating the differential risk of ADHD symptom dimensions and executive functioning (EF; time management, organization, motivation) to key occupational outcomes.

**Methods:**

Participants were adults (*N* = 100; 51% male) with ADHD working full-time between the ages of 19–30 (*M* = 26.61, SD = 2.28). Participants reported on their ADHD symptoms and EF as well as on five areas of occupational functioning (income, written warnings, boredom at work, satisfaction with coworkers and supervisors).

**Results:**

Over and above medication status, sex, and age, path analyses revealed hyperactivity/impulsivity and time management were the most consistent predictors of outcomes. Higher levels of each predictor were associated with lower income, more written warnings, greater boredom at work, as well as lower satisfaction with coworkers and supervisors. Executive function difficulties with organization and motivation were risk factors for all evaluated outcomes, except written warnings. Higher levels of inattention symptoms were associated with more written warnings and lower satisfaction with coworkers.

**Conclusion:**

The present study is one of the first to document the differential risks of ADHD symptoms and EF to a comprehensive set of occupational functioning outcomes. Future research is needed to replicate the present findings and expand this line of work to identify factors that may protect against these ADHD-related risks on workplace success.

## Introduction

Meaningful and successful employment is essential for high quality of life, well-being, and to obtain competitive wages/income [1]. Unfortunately, low professional obtainment and poor job satisfaction is common among individuals with attention-deficit/hyperactivity disorder (ADHD; [2]). Indeed, impaired work performance in ADHD starts early in adulthood [3], leading to an estimated 75% lower lifetime earnings as compared to non-ADHD peers [4]. To that end, research identifying ADHD-related occupational risks early (e.g., first years of employment), is sorely needed. However, for decades ADHD has predominantly been studied as a childhood disorder (for review, see [5]). Thus, while ADHD is a neurodevelopmental disorder associated with significant functional impairments across the lifespan [6], our understanding of how ADHD impacts occupational functioning in adulthood remains remarkably limited [7].

### ADHD Symptoms and Workplace Functioning

The vast majority of existing ADHD and workplace studies examine the disorder categorically to provide insights about occupational risks relative to non-ADHD peers (e.g., [4]). Importantly, however, ADHD is heterogeneous, and individuals present to the workplace with a range of ADHD-related strengths and weaknesses [7]. For example, early studies on ADHD and work, focused on the longitudinal outcomes of children with significant hyperactivity/impulsivity versus their non-hyperactive peers. In these studies, researchers found young adults with histories of elevated ADHD hyperactivity/impulsive symptoms, held similar jobs to their non-hyperactive counterparts ten years later [8, 9]. Later studies examining the effect of both dimensions of ADHD symptoms found inattention but not hyperactivity/impulsivity was associated with worse self-reported global indices of workplace functioning [10, 11]. These findings align with evidence that hyperactivity/impulsivity symptoms wane after childhood, and inattention symptoms become most impairing over time [12]. Similarly, evidence indicates childhood ADHD symptoms are associated with higher rates of high school dropout, while persisting inattention symptoms predict later occupational impairment [13]. Altogether, the reviewed evidence suggests that among ADHD symptom dimensions, inattention may be the strongest predictor of *global* workplace functioning.

Interestingly, however, when more *specific* measures of occupational functioning are utilized, hyperactivity/impulsivity symptoms are a significant predictor of work outcomes. For example, preliminary evidence indicates individuals with higher levels of hyperactivity/impulsivity are more likely to hold jobs with lower incomes, receive more disciplinary action at work, and are rated by supervisors as performing worse than their peers without ADHD [14–18]. Further, in a lab-based simulated work environment, individuals with ADHD who reported higher levels of hyperactivity/impulsivity symptoms rated higher levels of work-related boredom [19]. These findings suggest that hyperactivity/impulsivity symptoms impact specific aspects of work, including objective measures such as income and disciplinary action as well as more subjective experiences such as boredom at work. Collectively, the literature on ADHD symptoms to occupational functioning is sparse and highly mixed. Replication and further investigations are needed to clarify and confirm the associations between inattention and hyperactivity/impulsivity symptoms with occupational functioning.

### Time Management, Organization, and Motivation to Workplace Functioning

Executive functioning is another promising candidate for explaining the heterogeneity in occupational outcomes for individuals with ADHD. Barkley’s [20] unifying theory of ADHD characterizes executive functioning as the core underlying deficit in the disorder [20]. Executive functioning (EF) represents interrelated, higher-order cognitive processes such as working memory, inhibition, and problem solving that facilitate planning and goal-directed behaviors such as time management and planning [20, 21]. In addition, regulation of one’s motivational states using higher-order cognitive processes, are required for the successful engagement in and accomplishment of goal-directed behavior [20]. Indeed, behavioral manifestations of EF dysfunction are well-documented in ADHD, with time management, organization, and motivation most relevant for functioning [22, 23]. In addition, evidence-based ADHD interventions have been highly successful in assessing for and treating these behavioral EF impairments (e.g., [24, 25]). Thus, identifying and treating these EF deficits represent promising avenues of study and targets for occupational intervention in ADHD. However, EF deficits in ADHD have primarily been examined among children and adolescents (e.g., [23, 24]), and there is minimal understanding of the impact of organization, time management, and motivation deficits on workplace functioning for adults with ADHD.

Among the few studies that have examined EF deficits and occupational functioning, one study found individuals with ADHD experienced greater difficulties staying organized than non-ADHD controls [2]. However, the impact of these organizational difficulties on specific workplace demands were not examined. In another study, among individuals with ADHD, time management and motivation were associated with lower supervisor rated performance and more disciplinary actions at work [26]. In the same study, deficits in motivation were also associated with greater boredom at work, while problems with time management predicted greater workplace interpersonal difficulties [26]. Interestingly, organizational problems were not related to any occupational outcomes [26]. Altogether, these studies indicate problems with organization, time management, and motivation are present in working adults with ADHD. Moreover, further study on the effects of executive functioning on ADHD and workplace functioning is needed.

### ADHD and Relationships at Work

Finally, it will be important to understand how ADHD symptoms and executive functioning deficits differentially impact work-related interpersonal outcomes. Specifically, satisfaction with key work relationships, such as with supervisors and coworkers, is a critical aspect of occupational functioning. Satisfaction with work relationships may serve as a protective factor against poor work performance. However, qualitative studies indicate individuals with ADHD experience high levels of conflict with supervisors as well as isolation and loneliness from coworkers [10, 27, 28]. ADHD inattention symptoms and problems with organization, planning, and motivation may lead to difficulties performing job responsibilities; thus, resulting in less favorable supervisor perceptions and more strained supervisor-supervisee relationships. Similarly, hyperactivity/impulsivity symptoms (e.g., interrupting others) may result in fewer positive interactions with coworkers and supervisors. However, very little is known about predictors of work-related interpersonal outcomes for individuals with ADHD.

### Current Study

Limited research has examined the impact of ADHD symptom dimensions and executive functioning deficits to specific occupational outcomes [7]. This is an important gap to address, as understanding how prominent ADHD risks differentially impact occupational outcomes, can lead to the development of targeted and person-focused occupational interventions. To that end, the current study seeks to understand the differential impact of (1) inattention and hyperactivity/impulsivity symptoms and (2) executive functioning difficulties in time management, organization, and motivation on several key dimensions of occupational functioning impaired in ADHD (i.e., income, disciplinary action, boredom at work, as well as satisfaction with coworkers and supervisors). Given occupational impairment starts in early adulthood [3], with long-term financial ramifications, we selected to study individuals in their first 10 years of full-time employment. Based on the reviewed literature regarding ADHD and specific measures of occupational functioning, we hypothesize individuals with ADHD who have higher levels of hyperactivity/impulsivity symptoms, will hold lower income jobs, report more disciplinary actions at work, and be less satisfied with their relationship with supervisors. In addition, we hypothesize that lower EF motivation scores will be associated with greater boredom at work, as well as more problems with time management and higher levels of workplace interpersonal difficulties.

## Methods

### Participants

This study was approved by Rutgers University Institutional Review Board. Participants (*N* = 100; 51% male) were recruited from Amazon Mechanical Turk (MTurk; http://www.mturk.com) in the spring and summer of 2024 to complete an online survey on occupational functioning for individuals ages 19–30 with ADHD (*M* = 26.61, SD = 2.28). Participant characteristics for the current sample included 45% White/Non-Hispanic, 35% Black/African American, 18% White/Hispanic, and 2% Asian. Participant education included 1.9% completed high school, 5.6% some college/associate’s degree, 72.2% completed college, and 20.3% completed a graduate degree. Participants’ reported income ranged from $20,000 to $120,000 (*M* = 50,000 or less) on a categorical scale from $20,000 or less to $150,000 or more. Nineteen participants (19%) reported taking ADHD medications.

After completing consent, participants were administered a brief eligibility screening survey. Eligible participants were compensated $25 for completing the ADHD, EF, and occupational functioning surveys, which took approximately 50 min to complete. Study eligibility criteria included meeting full Diagnostic and Statistical Manual for Mental Disorders, Fifth Edition [29] criteria for ADHD inattentive or combined presentations, the most common presentations in adulthood [6]. Specifically, on the eligibility screener, participants had to endorse at least 5 current and 6 childhood inattention symptoms on the Barkley Adult ADHD Rating Scale-IV (BAARS; [30]). In addition, participants had to report working at least 20 h per week. Many individuals with ADHD struggle to find a 40-h per week job, thus including participants who work 20-h a week and above provides a more representative sample. Study exclusion criteria included (a) diagnoses of autism spectrum, bipolar, and schizophrenia disorder and/or (b) enrollment in higher education (defined as currently completing more than one college course). These criterions were selected, given the study’s focus on understanding occupational functioning, and because individuals engaged in college and the workforce will likely have different needs and have access to a wider range of free/low-cost resources. Seventy-four participants were screened out based on our exclusion criteria and/or the below described data quality and validity procedures.

### Data Quality and Validity Procedures

The study followed the highest level of methodological recommendations for MTurk surveys to ensure the accuracy of data (e.g., [31, 32]). Specifically, the study was restricted to workers in the United States who had at least a 95% approval rating and completed 100 or more approved Human Intelligence Tasks (HITS; tasks/surveys on Mturk). Further, to evaluate within person validity of responses, demographic variables (e.g., ADHD diagnostic status, ethnicity) were assessed multiple times within the survey with slight modifications [33]. In addition, the study design intentionally included open-ended responses to identify nonsensical or irrelevant responses (e.g., numerical values or symbols when asked about occupation; [33]), and these participants were removed. During data collection, participants who attempted to complete the eligibility survey/HIT more than once, were blocked from re-attempting the survey. Lastly, post data collection, study completion time was assessed for each participant, and had to be within the expected study range of 45 min to 1 h.

### Measures

#### ADHD Inattention and Hyperactivity/Impulsivity Symptoms

The Barkley Adult ADHD Rating Scale-IV (BAARS-IV; [30]) is a self-report measure that assesses the 18 DSM-5 symptoms of ADHD. Participants respond to each item on a 4-point Likert scale from 1 (sometimes) to 4 (very often). Internal consistencies in the present study were as follows: ADHD Inattention α = 0.70 and ADHD Hyperactivity/Impulsivity α = 0.83.

#### Executive Functioning

The Barkley Deficits in Executive Functioning Scale-Short Form (BDEFS-SF; [34])^.^is a 20-item, five subscale, rating scale to assess ADHD-related executive function difficulties. Items are rated using a four-point scale identical to the one described for the BAARS-IV. Higher scores indicate greater problems with executive functioning. Three subscales from the BDEFS-SF were used in the present study: self-management to time (Time), self-organization/problem solving (Organization), and self-motivation (Motivation). Study internal consistency for measures used were Time *α* = 0.70; Organization *α* = 0.70; and Motivation *α* = 0.70.

#### Job Satisfaction

The Job Satisfaction Survey (JSS; [35]) is a 36-item, nine subscales, self-report assessment of an employee’s attitudes and aspects of their job. Items are scored on a 6-point Likert scale from 1 = “Disagree very much” to 6 = “Agree very much,” with higher scores indicating more job satisfaction. For this study, two subscales were used: Coworkers (satisfaction with relationship with coworkers) and Supervision (satisfaction with one’s immediate supervisor). Internal consistency in the present study were Coworker *α* = 0.60 and Supervision *α* = 0.61.

#### Boredom at Work

The Dutch Boredom Scale (DUBS; [36]) is a 6-item self-report measure assessing experienced boredom at work. Items are assessed on a 5-point Likert scale from “never” to “always” to form a total score, with higher scores indicating greater experience of boredom at work. Internal consistency for the present study was *α* = 0.84.

#### Income and Disciplinary Action

Participants reported their approximate annual incomes on a categorical scale from $20,000 or less to $150,000 or more, with each category moving up by $10,000. For our measure of disciplinary action, participants were asked the number of times they received written disciplinary warnings within the past year from a well-published measure of occupational functioning (e.g., [37]). Specifically, this information was derived from the self-report measure Work History, which was developed as part of the Pittsburgh ADHD Longitudinal Study to compare the educational and occupational outcomes for young adults diagnosed with ADHD in childhood and followed into young adulthood [37].

### Analytical Plan

Multi-group path analyses were conducted in Mplus Version 8 [38]. Separate models were run to examine the effects of ADHD symptom severity (inattention, hyperactivity/impulsivity) and executive dysfunction difficulties with organization, time management, and motivation on five indicators of occupational functioning. Occupational outcomes were selected from the ADHD literature, which indicate individuals with ADHD are at-risk for lower income, higher levels of disciplinary action (e.g., written warnings), greater boredom at work, and more interpersonal difficulties with their supervisors and coworkers [10, 15, 17–19, 26]. Medication status, sex, and age were included in the model as covariates. Model fit was examined using several indicators: the comparative fit index (CFI), root mean square error approximation (RMSEA), and standardized root mean square residual (SRMR). CFI values > 0.90 suggest good fit. RMSEA values < 0.06 indicate good fit. SRMR values < 1.00 suggest good fit [38, 39].

## Results

### Preliminary Analyses

All independent/dependent variables were screened for univariate outliers, defined as values greater than 3 *SD* above/below the within-group mean. Two datapoints were identified as outliers and corrected to the most extreme value 3 *SD* above/below the within-group mean. Correlations between all covariates, independent, and dependent variables are shown in Table 1. There was a low percentage of missing data (1.31%) and data was missing completely at random (Little’s MCAR test: *χ2* = 38.59, *p* = 0.23). Full information maximum likelihood (FIML) was used to estimate missing data, which is appropriate for this low level of missing data [41]. FIML assumes all missing data is missing at random (MAR) and uses all observed information to estimate missing data as well as correct for small biases in the data by using the estimate of sample means [41].

**Table 1:** Means, standard deviations, and intercorrelations among primary study variables

	1	2	3	4	5	6	7	8	9	10	11	12	13
1. Age	–	0.39**	− 0.13	− 0.05	0.03	0.30**	0.29**	0.25*	− 0.08	0.18	0.22*	− 0.15	0.002
2. Sex	0.39**	–	− 0.10	− 0.19	− 0.15	0.06	− 0.03	0.03	0.10	− 0.03	− 0.01	− 0.02	0.001
3. Medication Status	− 0.13	0.10	–	− 0.13	− 0.03	− 0.01	− 0.005	0.07	0.31*	− 0.08	− 0.21*	0.02	0.08
4. Inattention	− 0.05	− 0.19	− 0.13	–	0.49**	0.22*	0.24*	0.21*	− 0.31*	0.24	0.18	− 0.24*	− 0.04
5. Hyperactivity/Impulsivity	0.03	− 0.15	− 0.03	0.49**	–	0.71**	0.73**	0.67**	− 0.32*	0.28**	0.55**	− 0.41**	− 0.41**
6. Time Management	0.30**	0.06	− 0.01	0.22*	0.71**	–	0.77**	0.80**	− 0.39**	0.27**	0.54**	− 0.45**	− 0.42**
7. Organization	0.29**	− 0.03	− 0.005	0.24*	0.73**	0.77**	–	0.80**	− 0.45**	0.18	0.55**	− 0.36**	− 0.38**
8. Motivation	0.25*	0.03	0.07	0.21*	0.67**	0.80**	0.80**	–	− 0.40**	0.22*	0.52**	− 0.39**	− 0.41**
9. Income	− 0.08	0.10	0.31*	− 0.31*	− 0.32*	− 0.39*	− 0.45**	− 0.40**	–	0.008	− 0.12	0.14	0.25
10. Written Warnings	0.18	− 0.03	−0.08	0.24*	0.28**	0.27**	0.18	0.22*	0.008	–	0.41**	− 0.35**	− 0.26
11. Boredom at Work	0.22*	− 0.01	− 0.21*	0.18	0.55*	0.54**	0.55**	0.52**	− 0.12	0.41**	–	− 0.62**	− 0.52**
12. Satisfaction with Coworkers	− 0.15	− 0.02	0.02	− 0.24*	− 0.41**	− 0.45**	− 0.36**	− 0.39**	0.14	− 0.35**	− 0.62**	–	0.43**
13. Satisfaction with Supervisors	0.002	0.001	0.08	− 0.04	− 0.41**	− 0.42**	− 0.38**	− 0.41**	0.25	− 0.26**	− 0.52**	0.43**	–
*M*	26.61	–	–	7.86	7.7	12.62	12.34	12.46	–	1.59	21.53	14.79	15.31
SD	2.28	–	–	1.41	1.87	2.06	2.16	2.16	–	0.10	4.64	2.60	2.69

*Indicates *p* < .05; ** indicates* p* ≤ .01. Participants reported income on a categorical scale and thus is not represented in this table. Income ranged from $20,000 to $120,000 (*M* = 50,000 or less) on a categorical scale from $20,000 or less to $150,000 or more

### Aim 1: Association Between ADHD Symptoms and Occupational Outcomes

**Fig. 1:**
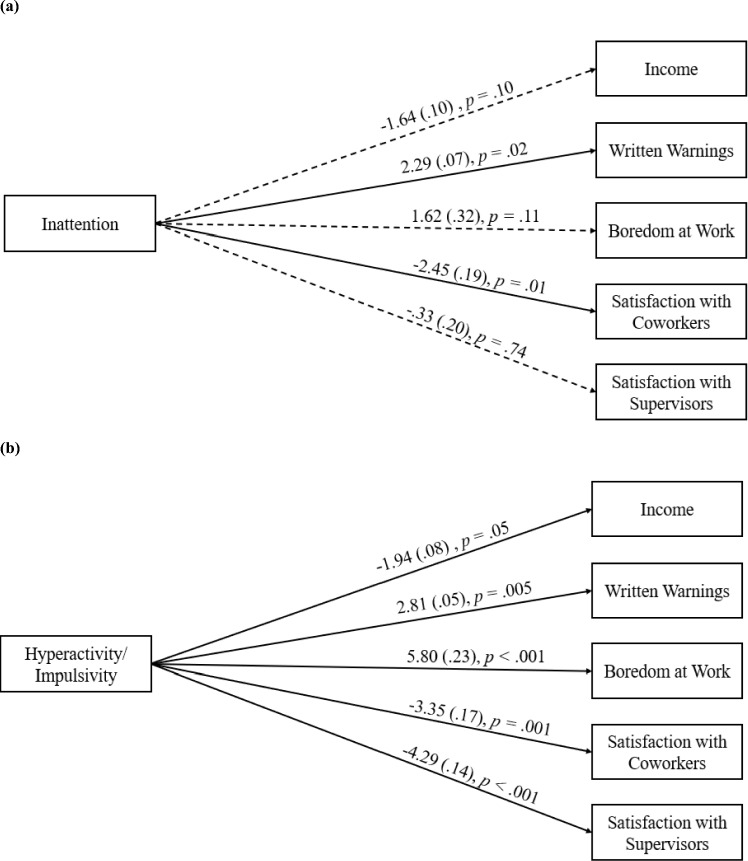
Association between ADHD symptoms and occupational outcomes. Relations between occupational outcomes and **a** inattention and **b** hyperactivity/impulsivity. Standardized coefficients shown outside parentheses. SEs are shown inside parentheses. Dashed paths are nonsignificant. Covariances are not included for readability

In two models, associations between ADHD inattention and hyperactivity/impulsivity symptoms were assessed to our five occupational outcomes (i.e., income, written warnings, boredom at work, satisfaction with coworkers, satisfaction with supervisors; Fig. 1). Model fit for both models were good on all three indices (all SRMR < 0.001; RMSEA < 0.001; CFI = 1.00). Results revealed higher inattention symptoms were associated with more written warnings in the past year (*β* = 2.29, *p* = 0.02) and less satisfaction with coworker relationships (*β* = − 2.45, *p* = − 0.01). No significant associations were found for inattention with income, boredom at work, and satisfaction with supervisors (*ps* > 0.10). In turn, higher ADHD hyperactivity/impulsivity symptoms were associated with lower income (*β* = −1.94, *p* = 0.05), more written warnings (*β* = 2.81, *p* = 0.005), greater boredom at work (*β* = 5.80, *p* < 0.001), as well as less satisfaction with relationships with coworkers (*β* = −3.35, *p* = 0.001) and supervisors (*β* = − 4.29, *p* < 0.001).

### Aim 2: Associations Between Time Management, Organization, and Motivation Deficits with Occupational Outcomes

**Fig. 2:**
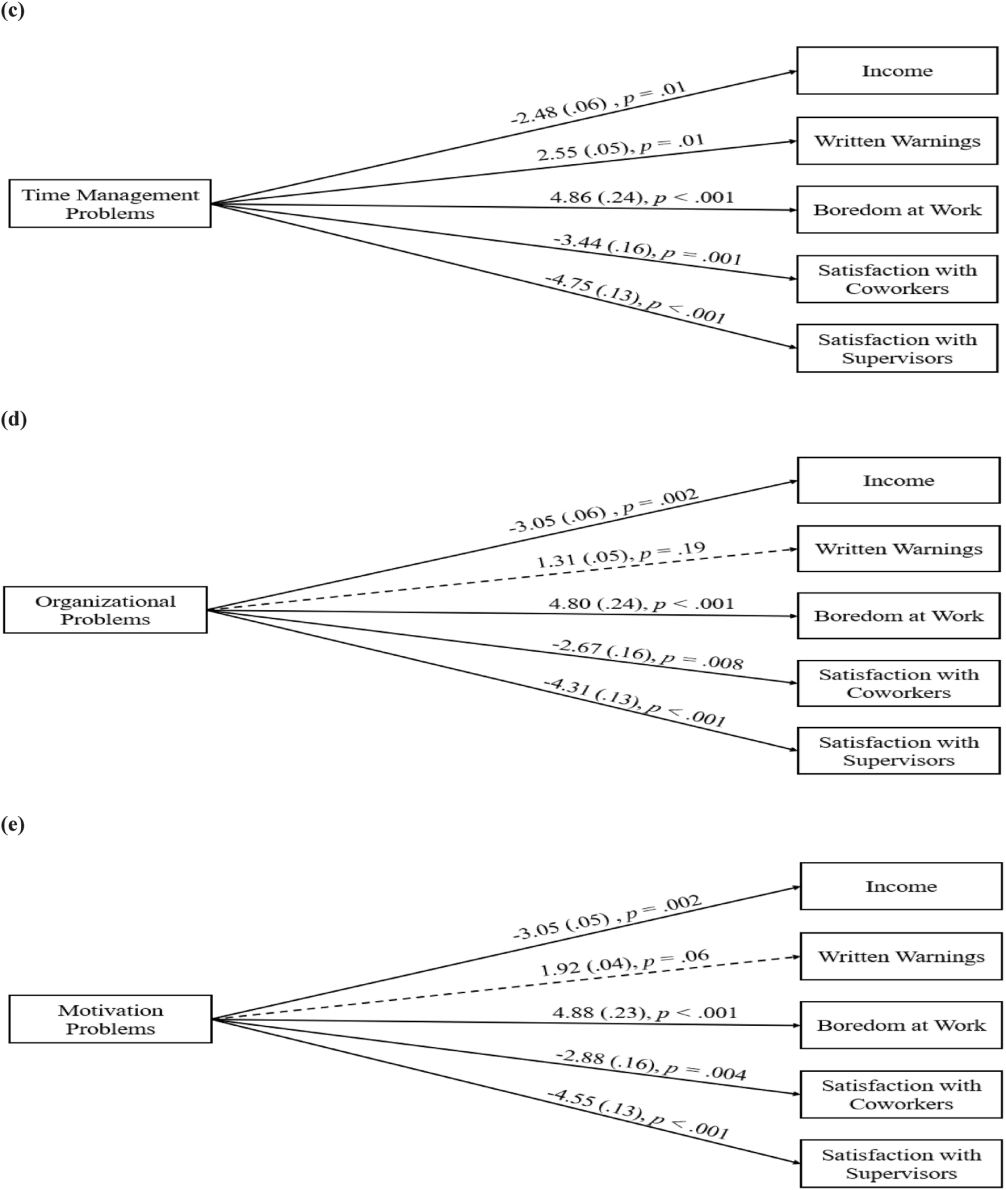
Association between executive functioning and occupational outcomes. Relations between occupational outcomes and **c** time management problems, **d** organizational problems, and **e** motivation problems. Standardized coefficients shown outside parentheses. SEs are shown inside parentheses. Dashed paths are nonsignificant. Covariances are not included for readability

In three models, associations between ADHD-related executive function (EF) difficulties with Time Management, Organization, Motivation, and the five occupational outcomes were assessed (Fig. 2). Model fit across models were good on all three indices (all SRMR < 0.001; RMSEA < 0.001; CFI = 1.00). Results revealed greater difficulties with time management was associated with lower income (*β* = − 2.48, *p* = 0.01), more written warnings (*β* = 2.55, *p* = 0.01), greater boredom at work (*β* = 4.86, *p* = 0.001), as well as lower satisfaction with coworkers (*β* = − 3.44, *p* = 0.001) and supervisors (*β* = − 4.75, *p* < 0.001). In addition, more difficulties with organization and motivation were associated with lower income (*β* = − 3.05, *p* = 0.002), greater boredom at work (*β* = 4.80 to 4.88, *ps* < 0.001), as well as lower satisfaction with coworkers (*β* = − 2.67 to − 2.88, *ps* < 0.008) and supervisors (*β* = − 4.31 to − 4.55, *ps* < 0.001). Neither organization nor motivation deficits were associated with more written warnings (*p* > 0.06).

## Discussion

The present study is one of the first to evaluate the differential effects of ADHD symptoms and executive function (EF) deficits in time management, organization, and motivation on multiple dimensions of occupational functioning. Among the ADHD symptoms and EF deficits examined, hyperactivity/impulsivity and time management problems were the most consistent predictors of occupational outcomes. These findings were robust to controls for medication status, sex, and age. Aligned with prior literature (e.g., [16, 19]), higher hyperactivity/impulsivity symptoms predicted lower income, more disciplinary action (i.e., written warnings), greater boredom at work, as well as lower satisfaction with one’s immediate supervisor. Unique to this study, we found higher hyperactivity/impulsivity was associated with lower satisfaction with coworkers.

Our findings replicate and underscore that, while hyperactivity/impulsivity symptoms may not predict global indicators of occupational functioning [10, 11], these symptoms have significant impact on social, achievement, and subjective experiences of work. Accordingly, research is needed to identify protective factors and work environments that promote success for individuals with high levels of hyperactivity/impulsivity. Typical work environments are highly structured and require long periods of time being quiet and seated (e.g., meetings and desk work). While little empirical work exists, authors have suggested individuals with ADHD may benefit from jobs that are more hands-on, socially oriented, as well as entrepreneurial and flexible [42–44]. Nonetheless, our work indicates that individuals with ADHD and high levels of hyperactive/impulsive symptoms may experience lower satisfaction with coworkers. This finding is aligned with prior work that indicates hyperactivity/impulsivity symptoms may predict lower peer relationships due to restless, impulsive, and intrusive behaviors being perceived as disruptive and/or annoying by peers [45, 46]. Future research could focus on characterizing working environments (e.g., peer climate) to determine if they moderate the association between hyperactivity/impulsivity and occupational outcomes.

Problems with time management also consistently predicted occupational functioning, including lower income, more disciplinary action, greater boredom at work, and lower satisfaction with coworkers and supervisors. Time management difficulties are prominent for children and adolescents with ADHD [22], and may be more impairing in adulthood as an individual is expected to be more self-sufficient in managing their schedule. For example, as youth with ADHD enter college, even those who are successful in high school often struggle in higher education due to increasing demands for independence, including managing one’s own schedule [25]. Often these difficulties stem from a loss of supports, such as reminders from parents, coordination of services across providers, or IEP plans [47]. Similarly, in the workplace, supervisors are frequently unaware of their employee’s ADHD status, and thus unable to provide needed supports. Further, even well-meaning supervisors who are aware of their employee’s ADHD diagnosis, are often reported to not understand how to support their employee [48]. Interestingly, qualitative evidence indicates employees with ADHD and difficulties with time management and organization, may rely on their coworkers to help them stay on-task such as by providing frequent reminders [27]. To that end, increasing evidence supports peer acceptance as protective for children and adolescents with ADHD (e.g., [49, 50]), and co-worker support may offer the same benefits for working adults with ADHD.

Our results further revealed executive function deficits in organization and motivation also predicted myriad occupational outcomes, including lower income, greater boredom at work, and lower satisfaction with coworkers and supervisors, but not disciplinary action. These findings are aligned with the broader ADHD literature, which indicate all three EF deficits (time management, organization, motivation) are significant predictors of achievement and social functioning [23]. Indeed, extant evidence-based ADHD treatments target all three EF deficits (e.g., organizational skills training; for review, see [25]). These interventions teach and reinforce use of tools, such as planners and breaking assignments into smaller deadlines [24, 25]—behavioral strategies that adults with ADHD report using at work as well [27]. Nonetheless, the developmental demands of the workplace and adulthood differs from the academic environment, which extant EF interventions for ADHD are developed for. For example, the workplace often has open ended and/or frequently changing deadlines, which differs from the school setting which has a curriculum/syllabus. Further, working adults frequently have competing demands, including caretaking roles (e.g., children, elderly parents) and financial responsibilities (e.g., mortgage, childcare, student debt). All these demands put significant pressure on the executive functioning of adults with ADHD.

Finally, inattention symptoms were associated with more disciplinary action and less satisfaction with coworkers, but not lower income, boredom at work, nor satisfaction with supervisors. Our findings are aligned with the ADHD literature, which consistently documents the impact of inattention symptoms on task performance and academic achievement (e.g., [12]), that in turn may lead to lower evaluations and more disciplinary action within the work setting. Further, one study found that while employees with ADHD have lower performance ratings (self, coworker, supervisor) than their non-ADHD peers, the lowest ratings were for their job-specific tasks [51]. Longitudinal research is needed to evaluate whether a failure to complete work tasks in a timely manner serves as a mediator between ADHD symptoms of inattention and disciplinary action. In regard to the impact of inattention symptoms on interpersonal functioning at work, individuals with ADHD inattention symptoms frequently experience difficulties with attending to details in conversation, assertiveness, and are prone to social withdrawal [52]. These social impairments may result in difficulties with building bonds and friendships among coworkers, which in turn, could result in individuals with ADHD lacking a sense of belonging and community at work and in less support from coworkers.

### Strengths, Limitations, and Future Study

There is very little research with reasonable sample sizes and diverse samples focused on the occupational functioning of adults with ADHD, and this study improves our understanding of specific risk factors. Our sample size was moderate and comprised of individuals from diverse ethnic backgrounds and incomes. Nonetheless, these findings need to be replicated in larger samples to ensure detection of small effects and generalizability of findings. Another strength was our use of measures specific to the workplace (e.g., Job Satisfaction Survey; Dutch Boredom Survey). Nonetheless, these measures were all self-report, and we relied on single measures of each construct (e.g., BDEFS for executive functioning). Thus, future research would benefit from utilizing multi-informant and objective measures (e.g., observation; tasks) of occupational and executive functioning (e.g., neuropsychological tests). Moreover, we selected to examine time management, organization, and self-motivation to work outcomes, given the extensive literature indicating these three EFs are prominent in ADHD, related to significant functional impairment, and the efficacy of our extant interventions at addressing these impairments [22–25]. Still, ADHD is highly heterogenous [53], and future research may benefit from examining additional indicators of executive functioning to work outcomes. Further, the data collected were cross-sectional, which prevents conclusions about directionality and limits the ability to evaluate moderators and mediators. In addition, the study defined full-time work as 20 or more hours per week and enrolled in no more than one academic course. We selected this inclusion criteria, as individuals with ADHD may struggle to find a traditional job with a 40-h work week and benefits. However, while these findings may reflect the reality of many working adults with ADHD, it may not be generalizable to a sample of 40 h per week workers only, who may have even higher demands for attention and self-regulation. Finally, we were unable to evaluate the impact of profession, industry, and job positions (e.g., managerial, employee) in the present study and this represents an important area of future study on ADHD and work.

## Conclusion

To our knowledge, the present study is one of the first to document the differential risks of ADHD symptoms and executive functioning to specific occupational outcomes. Collectively, study results indicate hyperactivity/impulsivity and time management difficulties were the most consistent predictors of occupational outcomes. Future research would benefit from exploring moderators and mediators of the risk to outcome association identified herein toward the development of occupational interventions for ADHD. It will also be important to research contextual factors, such as the types of job characteristics and environments that promote workplace success for individuals with ADHD (e.g., more hand-on, socially oriented, flexible; [42–44]). A good starting point may be to adapt existing evidence-based interventions targeting EF, organization, and time management to meet the demands of working adults with ADHD. Altogether, success in the workplace is critical for an individual’s well-being and self-sufficiency [1]. Future research is needed to replicate the present findings and expand this line of work to identify factors that may protect against these ADHD-related risks on workplace success.

## Data Availability

Data are  available from the corresponding author upon reasonable request.
